# Sex Differences, Depressive Symptoms, and Early Blood Pressure Control After Hospital Discharge in Cardiovascular Patients: A Prospective Observational Single-Center Study

**DOI:** 10.3390/life16091454

**Published:** 2026-08-31

**Authors:** Anastasija (Đorđe) Ilić, Aleksandar Petrović, Ana Balenović, Ivona Stanić Hadžalić, Aleksandra Milovančev, Andrej Preveden, Ana Marija Vejnović, Aleksandra Ilić

**Affiliations:** 1 Faculty of Medicine, University of Novi Sad, 21000 Novi Sad, Serbia; 2002stasa@gmail.com (A.I.); ap5382670@gmail.com (A.P.);; 2 Institute of Cardiovascular Diseases of Vojvodina, 21204 Sremska Kamenica, Serbia; 3 Clinic of Psychiatry, University Clinical Center of Vojvodina, 21000 Novi Sad, Serbia

**Keywords:** blood pressure control, cardiovascular hospitalization, depressive symptoms, anxiety symptoms, sex differences, psychological distress, secondary prevention

## Abstract

Background: Psychological distress is common among patients with cardiovascular disease and adversely affects secondary prevention. We investigated whether depressive and anxiety symptoms, identified by guideline-recommended ultra-brief screening instruments, were associated with blood pressure (BP) control after hospital discharge, with particular attention to sex. Data in very high-risk populations remain limited. Methods: In this prospective observational single-center study, 305 consecutive hospitalized cardiovascular patients (mean age 63.9 years, 62.6% men, 79.3% at very high cardiovascular risk) underwent screening for depressive and anxiety symptoms using the Patient Health Questionnaire-2 (PHQ-2) and Generalized Anxiety Disorder-2 (GAD-2). BP control was assessed at discharge and at routine follow-up after a median of 36.0 (32.5–39.5) days using ambulatory or home BP monitoring. Clinical, echocardiographic and psychosocial characteristics were analyzed. Results: Depressive and anxiety symptoms were identified in 31.1% and 29.2% of patients, respectively. Depressive symptoms were more prevalent in women. BP target achievement declined from 78.4% at discharge to 49.5% during follow-up. PHQ-2 ≥ 3 was independently associated with lower odds of achieving BP targets (OR 0.12, 95% CI 0.06–0.23; *p* < 0.001), together with overweight/obesity (OR 0.20, 95% CI 0.09–0.42; *p* < 0.001), while male sex was independently associated with better BP control (OR 2.06, 95% CI 1.12–3.77; *p* = 0.02). Female sex, overweight/obesity, chronic kidney disease, loneliness, and poor sleep quality were associated with PHQ-2 ≥ 3. Conclusions: Female sex and depressive symptoms were independently associated with a lower likelihood of achieving guideline-recommended BP targets during early post-discharge follow-up. These findings highlight the potential utility of brief psychological screening to identify patients at increased risk after discharge.

## 1. Introduction

Cardiovascular diseases (CVDs) are the leading cause of morbidity and mortality worldwide, with increasing recognition of the bidirectional relationship between cardiovascular and mental health. Depression and anxiety symptoms are highly prevalent among patients with CVD and are associated with worse clinical outcomes, reduced quality of life, and adverse disease progression. Conversely, CVD increases the risk of psychological distress [1,2,3,4].

Depressive symptoms are two to three times more common in patients with coronary artery disease than in the general population [5]. Following acute coronary syndrome, depression is associated with rehospitalization, poor medication adherence, and increased mortality [6,7,8]. Anxiety disorders are also prevalent in patients with heart failure, hypertension, and arrhythmias. Psychological distress may adversely affect cardiovascular outcomes through behavioral mechanisms, including poor medication adherence and unhealthy lifestyle behaviors, as well as biological pathways involving autonomic dysregulation, inflammation, and endothelial dysfunction [9,10].

The 2025 European Society of Cardiology (ESC) Consensus Statement recommends integration of psychological assessment into routine cardiovascular care, including the use of brief screening tools such as the Patient Health Questionnaire-2 (PHQ-2) and Generalized Anxiety Disorder-2 (GAD-2) for early identification of at-risk patients [11,12,13,14]. Increasing evidence indicates that sex differences influence not only cardiovascular disease presentation and outcomes but also the prevalence and clinical impact of psychological distress [15,16].

Although the association between psychosocial factors and CVD is well established [17,18,19,20], psychological assessment remains underutilized in hospitalized cardiovascular patients, where care is primarily focused on somatic, laboratory, and hemodynamic parameters, often overlooking psychological and psychosocial factors.

Previous studies have demonstrated a high burden of psychological distress in cardiovascular populations and its association with poorer recovery, treatment adherence, and secondary prevention outcomes [21,22,23]. Data from other high-risk European populations further support the clinical relevance of depression, anxiety, and sleep disorders in cardiovascular disease [24]. However, data from a very high-cardiovascular-risk region of Central and Southeast Europe remain limited. Central and Southeast European countries are characterized by a disproportionately high burden of cardiovascular disease, variable implementation of preventive cardiovascular care, and socioeconomic disparities [1] that may influence both psychological well-being and cardiovascular outcomes. These regional differences underscore the importance of generating population-specific evidence from this part of Europe.

Importantly, early after hospital discharge, adverse clinical outcomes have been observed, highlighting a vulnerable period for secondary prevention [25].

Therefore, this prospective observational study aimed to evaluate whether depressive and anxiety symptoms are associated with blood pressure control during early post-discharge follow-up in hospitalized cardiovascular patients. We also explored sex-related differences in psychological symptom burden and blood pressure control as clinically relevant components of contemporary cardiovascular secondary prevention.

## 2. Materials and Methods

### 2.1. Study Design and Population

This prospective observational single-center study included 305 consecutive adult patients (≥18 years) hospitalized at a tertiary cardiovascular center between December 2025 and April 2026 for various cardiovascular conditions, including coronary artery disease, acute myocardial infarction, heart failure, hypertension, arrhythmias, and cardiomyopathies. Consecutive enrollment was used to minimize selection bias. Participants underwent standardized screening for depressive and anxiety symptoms using guideline-recommended instruments and were prospectively followed during the vulnerable early post-discharge period to assess maintenance of blood pressure control. Only patients with complete data for all analyzed variables were included in the final analytical dataset. No imputation of missing data was performed.

Patients with severe cognitive impairment, inability to complete questionnaires or provide informed consent, critical clinical conditions (e.g., cardiogenic shock or mechanical ventilation), incomplete questionnaire data, or previously diagnosed psychiatric disorders were excluded.

**Figure 1:**
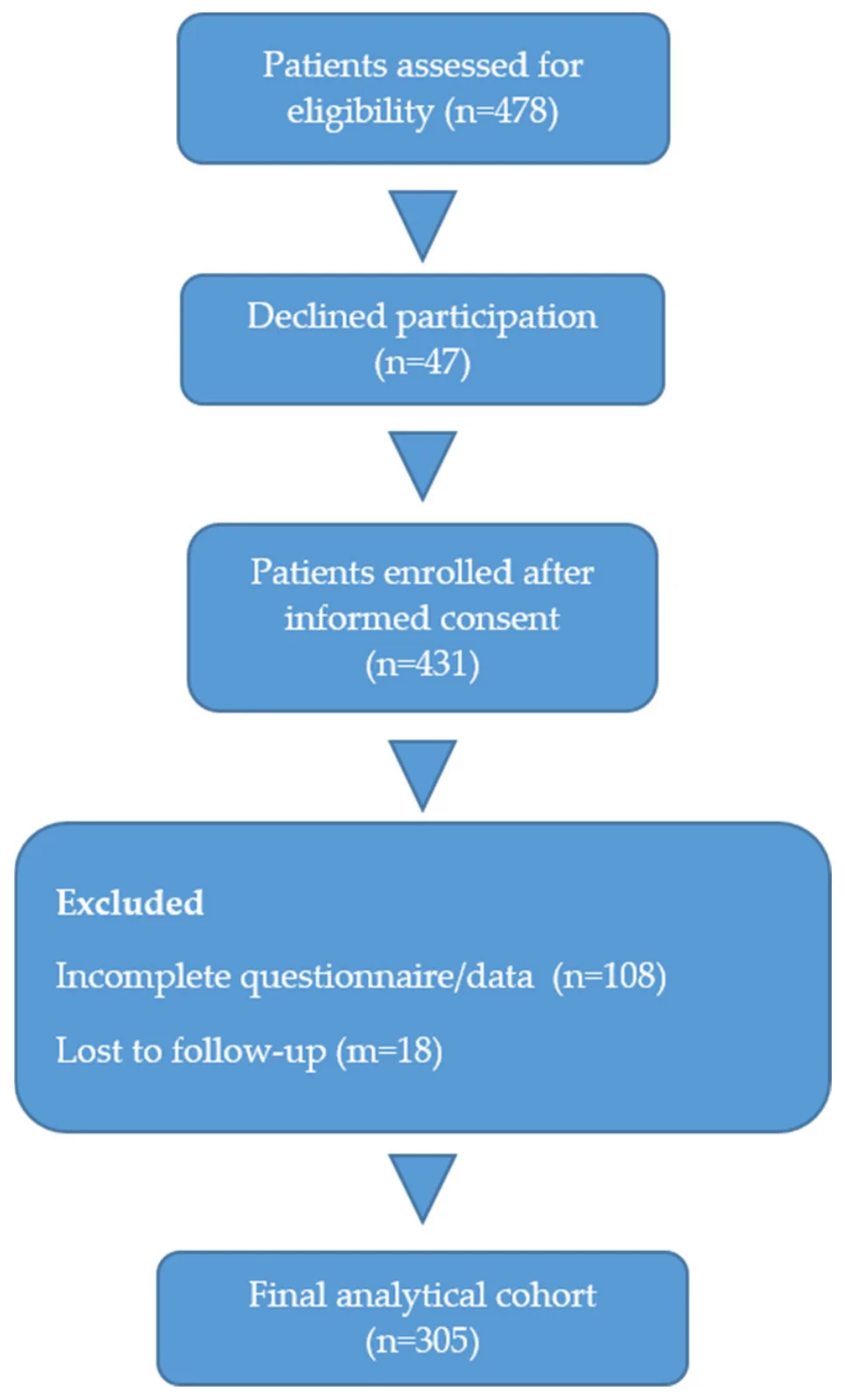
Study flow diagram.

Of 478 patients assessed for eligibility, 47 declined participation, 431 provided informed consent, 108 were excluded due to incomplete questionnaire/data, and 18 were lost to follow-up, resulting in a final analytical cohort of 305 patients (Figure 1).

### 2.2. Ethical Considerations

The study was approved by the institutional Ethics Committee (approval number: 2014-1/16) and was conducted in accordance with the Declaration of Helsinki. All participants provided written informed consent.

### 2.3. Data Collection

Demographic and clinical data were obtained from medical records and structured interviews, including age, sex, marital status, body mass index (BMI), type of admission, discharge diagnosis, cardiovascular risk factors, and comorbidities. Blood pressure and LDL cholesterol (LDL-C) values at discharge were recorded, and achievement of guideline-recommended blood pressure and LDL-C targets was assessed [26,27]. Blood pressure at discharge was measured according to routine in-hospital clinical practice using validated automated sphygmomanometers after clinical stabilization and was recorded from the final pre-discharge assessment. Additional exploratory analyses were performed to assess the potential influence of renal function parameters (serum creatinine, urea, and estimated glomerular filtration rate [eGFR]) and coronary artery disease status (CAD vs. non-CAD) on the observed associations. Estimated glomerular filtration rate (eGFR) was calculated using the Modification of Diet in Renal Disease (MDRD) equation [28].

### 2.4. Follow-Up Blood Pressure Assessment

Blood pressure control was assessed during routine outpatient follow-up visits at a median of 36.0 (IQR 32.5–39.5) days after discharge. Blood pressure values were obtained from either ambulatory blood pressure monitoring (ABPM) or home blood pressure monitoring (HBPM), depending on routine clinical practice and availability. Among the 305 patients included in the final analysis, 208 (68.2%) underwent ABPM and 97 (31.8%) underwent HBPM. ABPM measurements were performed using devices available in routine clinical practice. Since this observational study reflected real-world care, specific device models were not consistently available for all participants. Twenty-four-hour mean blood pressure values from ABPM recordings were used for analysis. For HBPM, patients were instructed to perform two measurements in the morning and two measurements in the evening for 7 consecutive days. The mean value of two consecutive measurements obtained during each measurement session was calculated, and the overall mean of available valid measurements was used for analysis. At the follow-up visit, patients were specifically asked whether any changes in antihypertensive therapy had occurred after hospital discharge. Patients who reported treatment modifications before follow-up were excluded from the final analysis; four such patients were among the 18 patients lost to follow-up. Blood pressure target achievement during follow-up was defined according to the 2024 ESC hypertension guidelines as blood pressure < 130/80 mmHg [26].

### 2.5. Echocardiographic Assessment

Transthoracic echocardiography was performed as part of routine clinical care. Left ventricular ejection fraction (LVEF) was recorded and categorized as <40%, 40–49%, or ≥50% [29]. Diastolic function was assessed using the E/e’ ratio [30].

### 2.6. Assessment of Depressive and Anxiety Symptoms

Depressive and anxiety symptoms were assessed using the Patient Health Questionnaire-2 (PHQ-2) and Generalized Anxiety Disorder-2 (GAD-2) scales [11]. The PHQ-2 and GAD-2 questionnaires were administered during index hospitalization before hospital discharge using translated versions available in clinical practice. Formal validation of these translated versions was not available, which is acknowledged as a limitation. A cutoff value of ≥3 was used to define clinically relevant symptoms, in line with validation studies favoring higher specificity [11,12,13,14]. The PHQ-2 and GAD-2 were used exclusively as screening instruments to identify depressive and anxiety symptoms and were not intended to establish clinical psychiatric diagnoses.

### 2.7. Psychosocial Factors and Adherence

Psychosocial variables were assessed using a study-specific structured questionnaire developed for the purposes of this study. The questionnaire included items on perceived loneliness/social isolation, financial difficulties, family/partnership and work-related problems, sleep quality, perceived family support, and patients’ understanding of the purpose of prescribed therapy. Medication adherence was assessed using a study-specific self-reported single-item question regarding missed prescribed medication before hospital admission. Adherence was categorized as adequate (never missed medication) or poor (occasional or frequent omission of prescribed medication).

### 2.8. Statistical Analysis

Statistical analyses were performed using SPSS version 17.0 (IBM Corp., Armonk, NY, USA). Continuous variables are presented as mean ± standard deviation (SD) or median (interquartile range [IQR]), as appropriate. Categorical variables are presented as frequencies and percentages. Between-group comparisons were performed using Student’s *t*-test or Mann–Whitney U test for continuous variables and the chi-square test or Fisher’s exact test for categorical variables, as appropriate. Univariable and multivariable logistic regression analyses were performed to identify independent predictors of achievement of blood pressure targets during early post-discharge follow-up and depressive symptoms (PHQ-2 ≥ 3). Variables with *p* < 0.10 in univariable analyses, together with clinically relevant variables, were considered for inclusion in multivariable models. For the blood pressure target achievement model, demographic, clinical, psychological, and psychosocial variables were considered. For the depressive symptoms model, demographic, clinical, and psychosocial variables were considered. Results are presented as odds ratios (ORs) with 95% confidence intervals (CIs). Model calibration was assessed using the Hosmer–Lemeshow goodness-of-fit test. A two-sided *p*-value < 0.05 was considered statistically significant.

## 3. Results

### 3.1. Baseline Characteristics

A total of 305 patients (mean (SD) age of 63.9 (12.1) years, 62.6% male) were included. Most patients were at very high cardiovascular risk (79.3%), with a high prevalence of hypertension (65.6%) and mean BMI of 29.1 (5.8) kg/m^2^. The most frequent comorbidities were atrial fibrillation (28.2%) and heart failure (22.0%). At discharge, LDL-C targets were achieved in 22.3% of patients, while blood pressure targets were achieved in 78.4%. During follow-up, performed after a median of 36.0 (IQR 32.5–39.5) days, blood pressure targets were achieved in 49.5% of patients. Emergency admissions accounted for 45.9% of the cohort (Table 1).

#### 3.1.1. Prevalence of Depressive and Anxiety Symptoms

Depressive symptoms (PHQ-2 ≥ 3) were identified in 31.1% of patients, whereas anxiety symptoms (GAD-2 ≥ 3) were present in 29.2% (Table 1).

#### 3.1.2. Distribution of Discharge Diagnoses

**Figure 2:**
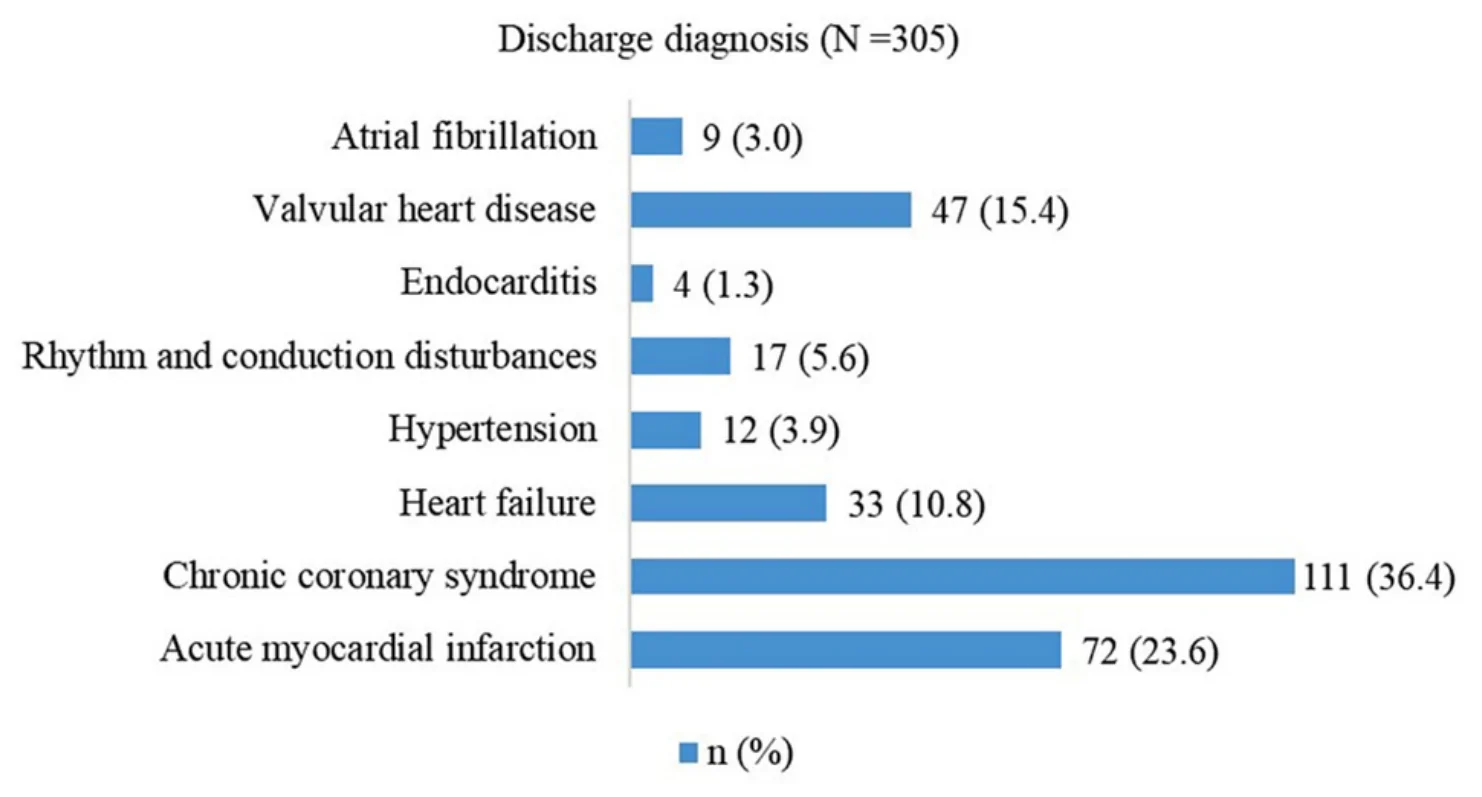
Distribution of discharge diagnoses (*N* = 305).

Coronary artery disease was the most frequent discharge diagnosis, accounting for 60.0% of cases (*n* = 183, 60.0%) when combining chronic and acute presentations, followed by valvular heart disease (*n* = 47, 15.4%) and heart failure (*n* = 33, 10.8%) (Figure 2).

### 3.2. Association of Demographic, Clinical, and Echocardiographic Characteristics with Depressive and Anxiety Symptoms

The proportion of male patients was lower among those screening positive for depressive symptoms compared with those without depressive symptoms (47.4% vs. 69.5%, *p* < 0.001). Patients screening positive for depressive symptoms (PHQ-2 ≥ 3) were less frequently married (54.7% vs. 72.4%, *p* = 0.02). They had a higher mean BMI (30.48 ± 6.51 vs. 28.54 ± 5.33 kg/m^2^, *p* = 0.01), a higher prevalence of BMI ≥ 25 kg/m^2^ (87.4% vs. 75.2%, *p* = 0.02), and higher systolic blood pressure (*p* = 0.03) and were less likely to achieve target blood pressure (68.4% vs. 82.9%, *p* = 0.01). Patients with PHQ-2 ≥ 3 also had higher E/e’ values (*p* = 0.03). No significant differences were observed in age, LDL cholesterol levels, LDL target achievement, or LVEF.

The proportion of male patients was lower among those screening positive for anxiety symptoms (GAD-2 ≥ 3) compared with those without anxiety symptoms (53.9% vs. 66.2%, *p* = 0.04), while no significant differences in those with GAD-2 ≥ 3 were observed in age, marital status, BMI, cardiovascular risk factor control, or echocardiographic parameters, including LVEF and E/e’ ratio (Table 2). Additional exploratory analyses were performed to assess the potential influence of renal function parameters and coronary artery disease status (CAD vs. non-CAD) on the observed associations. No significant differences in serum creatinine, urea, or eGFR were observed between PHQ-2 or GAD-2 categories. Similarly, neither depressive symptoms (PHQ-2 category, *p* = 0.13) nor achievement of the follow-up blood pressure target (*p* = 0.40) differed significantly between patients with and without coronary artery disease. Patients with eGFR < 60 mL/min/1.73 m^2^ more frequently achieved target blood pressure at follow-up than those with eGFR ≥ 60 mL/min/1.73 m^2^ (60.4% vs. 44.8%, *p* = 0.01).

### 3.3. Psychosocial Factors and Medication Adherence

Psychosocial stressors were more frequently reported among patients screening positive for depressive and anxiety symptoms (Table 3). Loneliness was markedly increased in both PHQ-2 ≥ 3 and GAD-2 ≥ 3 groups (88.4% and 68.5%, respectively; *p* < 0.001), along with higher rates of financial, family/relationship, and work-related problems. Poor sleep quality was also more frequent among symptomatic patients (*p* < 0.001 for both groups).

Self-reported poor medication adherence was more common in patients with depressive (80% vs. 48.1%, *p* < 0.001) and anxiety symptoms (71.9% vs. 52.3%, *p* = 0.02). Depressive symptoms were additionally associated with poorer understanding of treatment purpose (*p* = 0.02) and reduced family support (23.2% vs. 5.7%, *p* < 0.001).

### 3.4. Multivariable Analyses

In multivariable logistic regression analyses, female sex, overweight/obesity, and depressive symptoms (PHQ-2 ≥ 3) were independently associated with lower odds of achieving the target blood pressure at follow-up. Factors independently associated with depressive symptoms included female sex, BMI ≥ 25 kg/m^2^, loneliness, and poor sleep quality. Chronic kidney disease was also associated with depressive symptoms; however, this finding should be interpreted cautiously given the small number of patients with chronic kidney disease and the wide confidence interval of the estimate (Table 4).

Multivariable models demonstrated good calibration according to the Hosmer–Lemeshow goodness-of-fit test (blood pressure control model: χ^2^ = 4.541; *p* = 0.474; depressive symptoms model: χ^2^ = 12.521, *p* = 0.129).

## 4. Discussion

A major finding of the present study is the substantial decline in blood pressure control shortly after hospital discharge. Although blood pressure targets were achieved in the majority of patients at discharge, nearly half of the cohort failed to maintain guideline-recommended blood pressure levels during early outpatient follow-up. These findings highlight the vulnerability of the early post-discharge period and emphasize the gap between in-hospital optimization and long-term implementation of secondary prevention measures in routine clinical practice.

Importantly, depressive symptoms were independently associated with lower odds of achieving blood pressure targets after discharge. Our findings are consistent with previous evidence showing that depressive symptoms and poor medication adherence are associated with suboptimal blood pressure control in patients at high cardiovascular risk. In contrast to earlier studies based on documented psychiatric diagnoses [23], we used systematic PHQ-2 screening recommended by ESC guidelines, allowing standardized identification of at-risk patients.

To our knowledge, this is among the first prospective real-world studies from the Central and Southeast European region—characterized by a particularly high cardiovascular disease burden [1]—to evaluate the association between brief psychological screening and early post-discharge blood pressure control in hospitalized cardiovascular patients.

Approximately one-third of patients screened positive for depressive or anxiety symptoms. However, depressive symptoms demonstrated stronger and more consistent associations with adverse clinical and behavioral outcomes than anxiety symptoms. While previous studies have linked depression to adverse cardiovascular outcomes [25], our findings extend this evidence by demonstrating that depressive symptoms identified using the ultra-brief PHQ-2 instrument were independently associated with early loss of blood pressure control during the vulnerable post-discharge period. Because the PHQ-2 requires less than one minute to complete, these findings suggest that even ultra-brief psychological screening may help identify patients who warrant closer follow-up after hospital discharge. The observed associations are consistent with previous studies demonstrating the prognostic and functional relevance of psychological distress in cardiovascular populations [17,18,19,20,23].

Patients with depressive symptoms also demonstrated poorer self-reported medication adherence, impaired sleep quality, greater loneliness, and poorer understanding of prescribed therapy. These findings are consistent with previous evidence showing that depression and psychosocial stressors impair adherence to cardiovascular therapy and self-care behaviors [7,8,26,31,32], highlighting the multifactorial nature of this problem. Although medication adherence was not included as an independent predictor in the blood pressure model, its strong association with depressive symptoms suggests a potential behavioral pathway contributing to suboptimal blood pressure control, whereby psychological factors influence cardiovascular outcomes through behavioral pathways, including poor medication adherence and unhealthy lifestyle behaviors, as well as biological mechanisms such as autonomic dysregulation [3,4]. Recent evidence further suggests that mental disorders may themselves contribute to cardiovascular disease development, emphasizing the bidirectional relationship between psychological and cardiovascular health [33,34].

In contrast, anxiety symptoms showed weaker and less consistent associations with clinical and behavioral outcomes, suggesting a more context-dependent role in hospitalized cardiovascular patients. Although anxiety is also common among patients with cardiovascular disease and has been associated with adverse outcomes in previous studies [9,10,11,12], its clinical impact may vary depending on symptom severity, underlying disease characteristics, and the presence of coexisting depressive symptoms. The stronger associations observed for depressive symptoms in our cohort may reflect their closer relationship with treatment adherence, self-care behaviors, and sustained engagement in secondary prevention strategies.

Sex differences represented another clinically important finding of the present study. Women demonstrated a higher prevalence of both depressive and anxiety symptoms, consistent with previous cardiovascular literature [21,22,35], and were less likely to achieve target blood pressure during follow-up. Importantly, female sex remained independently associated with lower likelihood of blood pressure target achievement alongside depressive symptoms and overweight/obesity. These findings are consistent with previous cardiovascular literature suggesting greater psychosocial burden among women with cardiovascular disease [15]. However, whether sex modifies the association between depressive symptoms and blood pressure control requires further investigation. We also observed associations between psychological distress and adverse cardiometabolic profiles, including higher body mass index and higher E/e′ values. Although previous studies have suggested biologically plausible links between depressive symptoms and myocardial remodeling through neurohormonal and inflammatory pathways [3], this finding should be interpreted cautiously because E/e′ is influenced by multiple clinical factors, including blood pressure, volume status, obesity, renal function, and heart failure severity. Accordingly, no causal or mechanistic inference can be drawn from these cross-sectional associations. Nevertheless, our findings are consistent with previous reports describing an association between depressive symptom burden and diastolic dysfunction [9]. Whether ultra-brief screening instruments such as the PHQ-2 can help identify patients with early cardiac functional abnormalities requires confirmation in larger prospective studies.

The higher prevalence of overweight and obesity among patients with depressive symptoms further supports the clustering of psychological and metabolic risk factors, consistent with the concept of psychocardiometabolic interaction in which behavioral and biological mechanisms mutually reinforce cardiovascular risk [4,6,26].

Patients with chronic kidney disease (CKD) represent another vulnerable subgroup. In the present study, CKD was associated with depressive symptoms in multivariable analysis; however, this finding should be interpreted cautiously due to the small number of patients with CKD and the wide confidence interval of the estimate. This observation is in agreement with previous evidence linking CKD with depression and adverse cardiovascular outcomes [36]. In contrast, exploratory analyses of serum creatinine, urea, and eGFR did not demonstrate significant differences according to PHQ-2 status, likely reflecting the distinction between a chronic clinical diagnosis and single measurements of renal function. Interestingly, patients with eGFR < 60 mL/min/1.73 m^2^ more frequently achieved target blood pressure during follow-up, possibly reflecting closer clinical surveillance and more intensive antihypertensive management rather than a protective effect of impaired renal function itself. These exploratory findings should therefore be interpreted cautiously.

Given the heterogeneous nature of the study population, disease-specific differences cannot be excluded and should be investigated in larger prospective multicenter cohorts.

From a clinical perspective, our findings are consistent with the potential role of brief psychological screening tools, such as the PHQ-2 and GAD-2, in routine cardiovascular care, in accordance with recent ESC consensus recommendations [11]. Routine screening may facilitate early identification of patients at increased risk of suboptimal secondary prevention during the vulnerable post-discharge period, enabling targeted multidisciplinary interventions including adherence support, lifestyle counseling, psychosocial management, and closer follow-up. Particular attention may be warranted for women and for patients with combined psychological and cardiometabolic risk factors. Collectively, these findings support the implementation of sex-sensitive and psychologically informed approaches to cardiovascular secondary prevention.

### 4.1. Study Strengths

This study provides prospective real-world data on psychological distress in hospitalized cardiovascular patients from a very high-cardiovascular-risk region of Central and Southeast Europe. By combining systematic ESC-recommended psychological screening, cardiovascular risk factor assessment, medication adherence evaluation, echocardiographic parameters, and early post-discharge blood pressure follow-up, it offers a comprehensive assessment of the interplay between mental health and cardiovascular secondary prevention in routine clinical practice.

### 4.2. Study Limitations

Several limitations should be acknowledged. This was a single-center observational study conducted in a tertiary cardiovascular hospital, which may limit the generalizability of the findings to other healthcare settings and patient populations. Depressive and anxiety symptoms were assessed using validated screening instruments (PHQ-2 and GAD-2) rather than structured psychiatric interviews. Therefore, the present findings relate to depressive and anxiety symptoms identified by screening rather than to clinically confirmed psychiatric disorders. Although PHQ-2 and GAD-2 are recommended by the recent ESC Clinical Consensus Statement as feasible tools for routine cardiovascular practice, they do not establish clinical psychiatric diagnoses. The PHQ-2 and GAD-2 questionnaires were administered using translated versions available in routine clinical practice. However, formally validated Serbian-language versions were not available at the time of the study, which may have introduced some degree of measurement error. Medication adherence and psychosocial variables were assessed using study-specific structured self-report questions rather than validated instruments. Therefore, the results may have been influenced by reporting and recall bias, and some degree of misclassification of adherence and psychosocial factors cannot be excluded. Furthermore, post-discharge medication adherence was not prospectively assessed. Therefore, the potential influence of adherence patterns during follow-up on blood pressure control cannot be fully evaluated. Blood pressure follow-up was based on either ABPM or HBPM according to routine clinical practice, introducing some methodological heterogeneity. Although both methods provide clinically relevant out-of-office blood pressure estimates, differences in measurement approach and data acquisition may have introduced additional variability. Lipid levels were not systematically reassessed after discharge, precluding evaluation of the relationship between psychological distress and longitudinal LDL-C target achievement. Although exploratory analyses of renal function (serum creatinine, urea, and eGFR) did not demonstrate significant differences according to PHQ-2 status, residual confounding related to renal function, obesity, heart failure severity, volume status, and other factors influencing E/e′ cannot be excluded. Furthermore, the small number of patients with chronic kidney disease resulted in limited statistical power and reduced robustness of analyses involving renal comorbidity. The multivariable analysis adjusted for demographic and clinical variables, yet residual confounding from baseline blood pressure and other post-discharge factors remains possible. The relatively short follow-up period also limits the assessment of longer-term blood pressure control trajectories and the potential sustained impact of psychological factors on cardiovascular risk management. Finally, the cohort included a heterogeneous spectrum of cardiovascular diagnoses, reflecting routine clinical practice and contemporary ESC recommendations supporting psychological assessment across diverse cardiovascular populations. Although coronary artery disease represented approximately 60.0% of the study population, disease-specific subgroup analyses were not performed because the sample size within individual diagnostic categories was insufficient. Consequently, potential heterogeneity in the association between depressive symptoms and blood pressure control across different cardiovascular conditions cannot be excluded. Because the study population consisted of patients hospitalized in a cardiology department, the findings cannot be directly extrapolated to patients undergoing cardiac surgery or receiving perioperative cardiovascular care.

Finally, the observational design precludes conclusions regarding causality.

## 5. Conclusions

Female sex and screen-positive depressive symptoms were independently associated with lower odds of achieving guideline-recommended blood pressure control during the vulnerable early post-discharge period. Depressive and anxiety symptoms were highly prevalent among hospitalized cardiovascular patients. These findings underscore the potential value of brief psychological screening in cardiovascular care and highlight the early post-hospitalization period as a critical window for optimizing secondary prevention strategies. The simplicity and feasibility of these screening tools may facilitate their incorporation into routine clinical practice.

Future prospective multicenter studies should incorporate comprehensive psychiatric assessment alongside validated screening instruments, as well as validated medication adherence questionnaires, to better define the relationship between psychological symptoms, treatment adherence, and post-discharge blood pressure control in patients with cardiovascular disease. Such studies may help to define optimal strategies for integrating psychological screening into comprehensive cardiovascular risk management.

## Figures and Tables

**Table 1:** Demographic and clinical characteristics of the study population.

Parameter	Value
***Demographic characteristics***	
Age (years), mean (SD)	63.94 (12.06)
Male sex, *n* (%)	191 (62.6)
Married, *n* (%)	204 (66.9)
***Cardiovascular risk factors***	
Hypertension, *n* (%)	200 (65.6)
Hyperlipidemia, *n* (%)	138 (45.2)
Diabetes, *n* (%)	81 (26.6)
Smoking, *n* (%)	100 (32.8)
BMI, mean (SD)	29.14 (5.78)
BMI category ≥ 25 kg/m^2^, *n* (%)	241 (79.0)
***Comorbidities***	
Chronic obstructive pulmonary disease, *n* (%)	15 (4.9)
Chronic kidney disease, *n* (%)	8 (2.6)
Arrhythmias, *n* (%)	62 (20.3)
Atrial fibrillation, *n* (%)	86 (28.2)
Heart failure, *n* (%)	67 (22.0)
Prior myocardial infarction, *n* (%)	41 (13.4)
Prior stroke, *n* (%)	12 (3.9)
***Clinical parameters at discharge***	
LDL-C (mmol/L), mean (SD)	2.74 (1.11)
Systolic BP (mmHg), mean (SD)	130.82 (23.25)
Diastolic BP (mmHg), mean (SD)	77.66 (13.45)
***Blood pressure at follow-up***	
Systolic BP (mmHg), mean (SD)	136.29 (15.95)
Diastolic BP (mmHg), mean (SD)	84.33 (9.50)
***Echocardiographic parameters***	
LVEF (%), mean (SD)	49.13 (12.86)
LVEF < 40%, *n* (%)	65 ((21.3)
LVEF 40–49%, *n* (%)	58 19.0)
LVEF ≥ 50%, *n* (%)	182 (59.7)
E/e’ ratio, mean (SD)	11.79 (4.97)
***Type of admission***	
Emergency admission, *n* (%)	140 (45.9)
***Cardiovascular risk and target achievement***	
Very high cardiovascular risk, *n* (%)	242 (79.3)
LDL-C target achieved at discharge, *n* (%)	68 (22.3)
BP target achieved at discharge, *n* (%)	239 (78.4)
BP target achieved at follow-up, *n* (%)	151 (49.5)
***Screening results***	
PHQ-2 ≥ 3, *n* (%)	95 (31.1)
GAD-2 ≥ 3, *n* (%)	89 (29.2)

Values are presented as mean (SD) or number (percentage), as appropriate. Legend: BMI, body mass index; LDL-C, low-density lipoprotein cholesterol; BP, blood pressure; LVEF, left ventricular ejection fraction; E/e’, the ratio of early diastolic mitral inflow velocity (E) to early diastolic mitral annular velocity (e’); PHQ-2, Patient Health Questionnaire-2; GAD-2, Generalized Anxiety Disorder-2.

**Table 2:** Association of demographic, clinical, and echocardiographic characteristics with depressive and anxiety symptoms.

Parameter	PHQ-2 < 3	PHQ-2 ≥ 3	P1-Value	GAD-2 < 3	GAD-2 ≥ 3	P2-Value
**Demographic characteristics**						
Male sex, *n* (%)	146 (69.5)	45 (47.4)	<0.001	143 (66.2)	48 (53.9)	0.04
Age (years), mean (SD)	63.57 (11.87)	64.77 (12.50)	0.42	64.59 (11.78)	62.38 (12.64)	0.15
**Marital status, *n* (%)**						
Married	152 (72.4)	52 (54.7)	0.02	149 (69.0)	55 (61.8)	0.51
Widower/widow	13 (6.2)	7 (7.4)		13 (6.0)	7 (7.9)	
Single	13 (6.2)	10 (10.5)		17 (7.9)	6 (6.7)	
Divorced	32 (15.2)	26 (27.4)		37 (17.1)	21 (23.6)	
**Risk factors**						
BMI (kg/m^2^), mean (SD)	28.54 (5.33)	30.48 (6.51)	0.01	29.23 (6.01)	28.94 (5.23)	0.69
BMI ≥ 25, *n* (%)	158 (75.2)	83 (87.4)	0.02	158 (75.2)	83 (87.4)	0.68
LDL-C (mmol/L), mean (SD)	2.78 (1.17)	2.65 (0.99)	0.29	2.75 (1.13)	2.74 (1.08)	0.97
Systolic BP (mmHg), mean (SD)	128.70 (21.16)	135.53 (26.83)	0.03	131.37 (22.04)	129.51 (26.04)	0.53
Diastolic BP (mmHg), mean (SD)	76.67 (12.07)	79.85 (16.01)	0.09	78.06 (12.28)	76.70 (16.05)	0.42
BP target achieved at discharge, *n* (%)	174 (82.9)	65 (68.4)	0.01	174 (80.6)	65 (73.0)	0.15
BP target achieved at follow-up, *n* (%)	136 (64.8)	15 (15.8)	<0.001	113 (52.3)	38 (42.7)	0.13
LDL-C target achieved, *n* (%)	51 (24.3)	17 (17.9)	0.21	48 (22.2)	20 (22.5)	0.96
**Echocardiographic parameters**						
LVEF < 40%, *n* (%)	38 (18.2)	27 (28.7)	0.12	45 (20.9)	20 (22.7)	0.92
LVEF 40–49%, *n* (%)	42 (20.1)	16 (17.0)		42 (19.5)	16 (18.2)	
LVEF ≥ 50%, *n* (%)	129 (61.7)	51 (54.3)		128 (59.5)	52 (59.1)	
LVEF, mean (SD)	49.96 (12.63)	47.28 (13.23)	0.09	48.95 (13.00)	49.56 (12.57)	0.71
E/e’ ratio, mean (SD)	11.38 (5.01)	12.70 (4.80)	0.03	11.71 (4.74)	12.01 (5.50)	0.63

Values are presented as mean (SD) or number (percentage), as appropriate. Legend: see Table 1; P1 indicates the comparison between participants with PHQ-2 < 3 and PHQ-2 ≥ 3; P2 indicates the comparison between participants with GAD-2 < 3 and GAD-2 ≥ 3.

**Table 3:** Psychosocial factors and treatment adherence according to PHQ-2 and GAD-2 screening results.

Parameter	PHQ-2 < 3 (*n* = 210)	PHQ-2 ≥ 3 (*n* = 95)	P1-Value	GAD-2 < 3 (*n* = 216)	GAD-2 ≥ 3 (*n* = 89)	P2-Value
**Feelings of loneliness**, *n* (%)			<0.001			<0.001
Never	135 (64.3)	11 (11.6)		118 (54.6)	28 (31.5)	
Occasionally/Often/Constantly	75 (35.7)	84 (88.4)		98 (45.4)	61 (68.5)	
**Financial problems**, *n* (%)			<0.001			<0.001
None	154 (73.3)	55 (57.9)		161 (74.5)	48 (53.9)	
Partial/Significant	56 (26.7)	40 (42.1)		55 (25.5)	41 (46.1)	
**Family/partnership problems**, *n* (%)			0.01			<0.001
None	172 (81.9)	60 (63.2)		177 (81.9)	55 (61.8)	
Partial/Significant	38 (18.1)	35 (36.8)		39 (18.1)	34 (38.2)	
**Work-related problems**, *n* (%)			0.03			0.02
None	109 (51.9)	36 (37.9)		109 (50.5)	36 (40.4)	
Partial/Significant	101 (48.1)	59 (62.1)		107 (49.5)	53 (59.6)	
**Sleep quality**, *n* (%)			<0.001			<0.001
Excellent/Good	181 (86.2)	64 (67.3)		182 (84.3)	63 (70.8)	
Poor/Very poor	29 (13.8)	31 (32.7)		34 (15.7)	26 (29.2)	
**Missing medication doses**, *n* (%)			<0.001			0.02
Never	109 (51.9)	19 (20.0)		103 (47.7)	25 (28.1)	
Sometimes/Often/Can’t remember	101 (48.1)	76 (80.0)		113 (52.3)	64 (71.9)	
**Understanding treatment purpose**, *n* (%)			0.02			0.69
Yes	172 (81.9)	64 (67.4)		170 (78.7)	66 (74.2)	
Partial/No	38 (18.1)	31 (32.6)		46 (21.3)	23 (25.8)	
**Family support**, *n* (%)			<0.001			0.16
Yes	198 (94.3)	73 (76.8)		196 (90.7)	75 (84.3)	
Partial/No	12 (5.7)	22 (23.2)		20 (9.3)	14 (15.7)	

Values are presented as number (percentage). Legend: see Table 1; P1 indicates the comparison between participants with PHQ-2 < 3 and PHQ-2 ≥ 3; P2 indicates the comparison between participants with GAD-2 < 3 and GAD-2 ≥ 3.

**Table 4:** Multivariable logistic regression analyses for blood pressure target achievement and depressive symptoms.

Parameter	BP Target Achievement OR (95% CI)	P1-Value	PHQ-2 ≥ 3 OR (95% CI)	P2-Value
Male sex	2.06 (1.12–3.77)	0.02	0.35 (0.18–0.66)	0.01
BMI ≥ 25 kg/m^2^	0.20 (0.09–0.42)	<0.001	4.50 (1.79–11.30)	0.01
Chronic kidney disease	—	—	7.95 (1.40–45.06)	0.02
PHQ-2 ≥ 3	0.12 (0.06–0.23)	<0.001	—	—
Sense of loneliness	—	—	5.87 (3.52–9.81)	<0.001
Good sleep quality	—	—	0.67 (0.50–0.90)	0.01

Values are presented as odds ratios (ORs) with 95% confidence intervals (CIs). Legend: see Table 1; P1 indicates *p*-values for the blood pressure target achievement model; P2 indicates *p*-values for the PHQ-2 ≥ 3 model.

## Data Availability

The data presented in this study are available on request from the corresponding author.

## Abbreviations

The following abbreviations are used in this manuscript:

BMI	Body mass index
BP	Blood pressure
CKD	Chronic kidney disease
CVD	Cardiovascular disease
E/e′	Ratio of early diastolic mitral inflow velocity (E) to early diastolic mitral annular velocity (e′)
ESC	European Society of Cardiology
GAD-2	Generalized Anxiety Disorder-2
HBPM	Home blood pressure monitoring
IQR	Interquartile range
LDL-C	Low-density lipoprotein cholesterol
LVEF	Left ventricular ejection fraction
MDRD	Modification of Diet in Renal Disease
OR	Odds ratio
PHQ-2	Patient Health Questionnaire-2
SD	Standard deviation
